# Effect of Biological Treatment in Uncontrolled Severe Chronic Rhinosinusitis with Polyps—A Real-Life Experience

**DOI:** 10.3390/biomedicines14061228

**Published:** 2026-05-29

**Authors:** Na Sun, Ziye Huang, Yu Zhan

**Affiliations:** Department of Otorhinolaryngology, Guanghua Hospital Affiliated to Shanghai University of Traditional Chinese Medicine, Shanghai 200052, China; huangziye1713@163.com (Z.H.); 13122232878@163.com (Y.Z.)

**Keywords:** CRSwNPs, mepolizumab, NPS, SNOT-22, smell, nasal obstruction

## Abstract

**Background**: The aim of this study was to evaluate the efficacy of mepolizumab as add-on therapy to intranasal corticosteroids (INCSs) for the treatment of severe, uncontrolled chronic rhinosinusitis with nasal polyps (CRSwNPs) in a real-life setting. **Methods**: This prospective observational study included 60 patients with severe uncontrolled CRSwNP who received mepolizumab. Follow-up assessments were performed at baseline (T0), 3 months (T1), and 6 months (T2). At each time point, patients underwent nasal endoscopy, completed the sinonasal outcome test-22 (SNOT-22), visual analogue scales (VAS) for smell, nasal obstruction and rhinorrhoea and facial pain. Nasal secretion and blood eosinophil counts (BECs) were also evaluated. The levels of eosinophil cationic protein (ECP) in nasal secretions were measured using an enzyme-linked immunosorbent assay (ELISA). **Results**: Both patient- and physician-derived outcome measures showed significant improvements from baseline to 3 months, and the benefits were maintained at 6 months. No major adverse events were reported. **Conclusions**: Mepolizumab was associated with improvements in nasal obstruction and sense of smell, based on both patient- and physician-derived outcome measures. However, due to the single-arm design and modest sample size, these findings should be considered hypothesis-generating rather than confirmatory.

## 1. Introduction

Chronic rhinosinusitis with nasal polyps (CRSwNPs) is a debilitating inflammatory disease of the paranasal sinuses characterized by a type 2 inflammatory endotype, often associated with elevated levels of interleukin-5 (IL-5) and peripheral and tissue eosinophilia [1]. A significant subset of patients remains severely symptomatic and uncontrolled despite maximal standard therapy, including systemic corticosteroids and endoscopic sinus surgery (ESS) [2]. This population experiences a severely impaired quality of life due to persistent nasal obstruction, anosmia, rhinorrhoea, and facial pain. The recognition of IL-5 as a pivotal cytokine in the differentiation, survival, and activation of eosinophils has established it as a key therapeutic target in type 2 inflammatory diseases [3,4].

Mepolizumab, a humanized monoclonal antibody targeting IL-5, has emerged as a promising therapeutic option by inhibiting eosinophilic inflammation [5]. While randomized controlled trials (RCTs) have established its efficacy in reducing nasal polyp size and improving symptoms [6,7], data from real-world settings are still evolving. Real-life studies are crucial to confirm RCT findings in heterogeneous patient populations encountering everyday clinical practice variations.

The primary objective of this real-life study was to evaluate the effectiveness of mepolizumab as an add-on therapy to intranasal corticosteroids (INCSs) in patients with severe, uncontrolled CRSwNPs, using a comprehensive set of both objective and patient-reported outcome measures over a 6-month period.

## 2. Materials and Methods

### 2.1. Patient Population

A prospective, single-centre, observational study was conducted from January 2025 to March 2026. All patients were consecutively enrolled into this cohort study. No selection was made based on disease severity, surgical history, disease duration, or asthma status. We collected data at baseline (before starting the biological treatment) (T0) and at subsequent follow-up visits [3 months (T1) and 6 months (T2)]. Before treatment initiation, baseline demographic and clinical data were obtained from electronic medical records and independently verified by two investigators: anthropometric and demographic information, surgical history, respiratory allergen sensitivity, active smoking status, and the number of oral corticosteroids (OCSs) short courses in the 12 months prior to enrollment. Definitions and classifications for key variables (e.g., allergen sensitivity, smoking habit, OCS courses) followed the EPOS 2020 guideline [1]. At each time point, patients were assessed by means of the sinonasal outcome test-22 (SNOT-22) (20), and by means of a nasal endoscopy.

Study participants were recruited and followed in the rhinological units of Guanghua Hospital Affiliated to Shanghai University of Traditional Chinese in Shanghai during the first 6 months after approval of mepolizumab. Inclusion criteria were defined according to the Guideline for diagnosis and treatment of chronic rhinosinusitis (2024) [8]:

(1) Age ≥ 18 years;
(2) diagnosis of severe CRSwNP, defined by a nasal polyp score (NPS) ≥ 5 and/or a SNOT-22 ≥ 50, with inadequate symptom control despite INCS use, receiving at least 2 cycles of systemic corticosteroid in the last year and/or having undergone one or more sinonasal surgeries [ESS];
(3) no planned sinonasal surgery during the study period;
(4) prior ESS was allowed (or not), but no surgery had been performed within at least 3 months before enrollment;
(5) administration of mepolizumab 100 mg, one sub-cutaneous injection every four weeks, indicated specifically for severe CRSwNP treatment as an add-on therapy to INCS as conventional treatment [9]. Patients were excluded if they received another biological treatment for CRSwNP within the 3 months before the start of the study.

Bilateral diffuse CRS with predominance of type 2 inflammation was evident in each patient. Criteria considered for type 2 endotyping were: history of elevated blood eosinophil counts (BEC) and/or high levels of eosinophil infiltrate in previous surgical biopsies [1,10].

Anthropometric and demographic data, surgical history, and respiratory allergens sensitivity in the previous 12 months [1] were collected before starting the treatment. At each timepoint, patients were assessed by means of SNOT-22 [11], and by means of a nasal endoscopy (using 0° rigid endoscope) to assess both NPS [12,13]. The sino-nasal symptoms were also collected using the visual analogue scale (VAS) scores for nasal obstruction (VAS-NO), smell (VAS-smell), rhinorrhoea (VAS-rhinorrhoea) and facial pain (VAS-facial pain) [14], and whenever comorbid asthma was present.

All patients continued their pre-existing background therapy with INCS and nasal irrigation throughout the study period. Patients had been using these treatments regularly for at least 3 months prior to enrollment. No additional standardization or protocol-mandated adjustments were applied to concomitant treatments, reflecting real-world clinical practice.

### 2.2. Sample Collection

Cytological smears and blood were collected from all patients 3 times at the beginning and after 3 and 6 months of treatment. Nasal tissue biopsy was not performed due to ethical and practical considerations. Instead, nasal secretions and blood samples were collected as surrogate markers, as previous studies have demonstrated that inflammatory mediators in these samples correlate with histopathological changes in sinonasal mucosa [15]. Furthermore, nasal cytology represents a simpler, non-invasive, and reproducible method for assessing local eosinophilia, and is particularly valuable for monitoring therapeutic response to biologics over time [16]. Nasal cytology smears were collected from the middle portion of the bilateral inferior turbinates and stained with eosin. Eosinophil counts were evaluated under light microscopy at 400× magnification and expressed as the mean number of cells per 50 high-power fields. Peripheral BECs were also measured at each visit. ECP concentrations in nasal secretions and supernatants were detected by ELISA, according to ELISA Kits (Wuhan Zhongminteck Biotechnology Co., Ltd., Wuhan, China), and the results were expressed as μg/mL.

### 2.3. Monitoring of Adverse Events and Treatment Adherence

During each follow-up visit, patients were interviewed regarding any adverse events (AEs), prescriptions for antibiotics or OCSs, and whether they had undergone revision ESS. Adherence to the biologic treatment regimen was reviewed, along with compliance to concomitant standard care involving INCS and nasal irrigation.

### 2.4. Ethics and Consent

This study complied with all applicable laws regarding patient privacy. Where data had already been collected in registries, no direct subject contact or primary collection of individual human subject data occurred. Where additional patient data were required, individual consent was obtained prior to the use of preexisting patient data. Informed consent was confirmed from all data cohorts. The consent form was approved by the Ethics Committee of Guanghua Hospital affiliated to Shanghai University of Traditional Chinese Medicine (approval number: 2025-K-76), which was compliant with the General Data Protection Regulation and any local requirements. Patients who did not provide informed consent were excluded from this analysis. Informed consent on personal data collection and use for research purposes was obtained from each subject before starting mepolizumab treatment.

### 2.5. Statistical Analyses

Statistical analyses were performed with GraphPad Prism VI for Macintosh Version 8.4.3 (GraphPad Software Inc., San Diego, CA, USA).

Measurement data are expressed as mean ± standard deviation (Mean ± SD). If it conforms to the normal distribution, the *t*-test is used for comparison between two groups, and one-way analysis of variance (ANOVA) is used for comparison among three groups. Tukey’s correction is used for multiple comparisons. Pairwise comparisons of differences between groups are performed by the LSD-*t* test. For measurement data that do not meet the normal distribution, the Kruskal–Wallis non-parametric test is used. Enumeration data are expressed as cases (%) and the χ^2^ test is used. Values will be considered statistically significant if *p*-values < 0.05.

## 3. Results

Based on the effect size of the pre-experiment, we planned to recruit 80 cases. However, due to some patients interrupting the treatment, there were ultimately 60 eligible cases for follow-up. A total of 60 patients with CRSwNP were treated with mepolizumab (mean age 40.5 ± 11, 100 mg every 28 days). There were almost 3% more males than females in our cohort, an average disease duration of more than 10 years, and the majority of patients had undergone one or two ESS in the past. All patients had a significantly higher BECs. Baseline characteristics are listed in Table 1.

**Figure 1:**
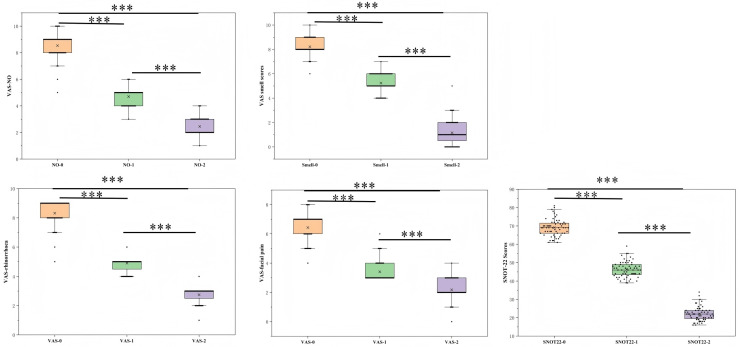
Patient-derived outcome measure changes during the study period. The sino-nasal symptoms were collected using the VAS scores for nasal obstruction (VAS-NO), smell (VAS-smell), rhinorrhoea, (VAS-rhinorrhoea) and facial pain (VAS-facial pain). Data are presented as the means ± standard deviation (SD), significance calculated using mixed effect analysis with Tukey’s multiple comparisons test, ***: *p* < 0.001. SNOT-22: Sinonasal Outcome Test-22; NO: Nasal Obstruction; VAS: Visual Analogue Scale; 0—before treatment, 1—after 3 months of treatment, 2—after 6 months of treatment. Orange, green, and purple boxes represent baseline (T0), 3 months (T1), and 6 months (T2).

**Figure 2:**
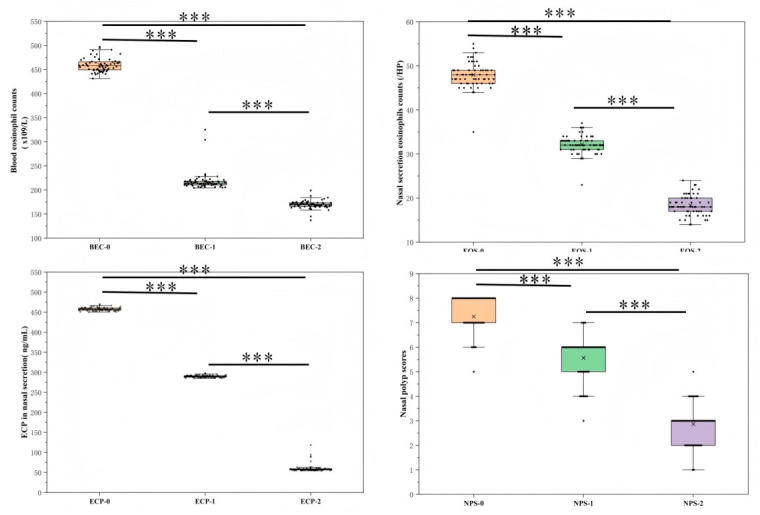
Physician-derived outcome measure changes during the study period. Data are presented as the means ± standard deviation (SD), significance is calculated using mixed effect analysis with Tukey’s multiple comparisons test, ***: *p* < 0.001. Eos: Eosinophils cells in nasal secretion sample; Bec: Blood eosinophil counts (cells × 10^9^/L); NPS: Bilateral Nasal Polyp Scores; 0—before treatment, 1—after 3 months of treatment, 2—after 6 months of treatment. Orange, green, and purple boxes represent baseline (T0), 3 months (T1), and 6 months (T2).

**Figure 3:**
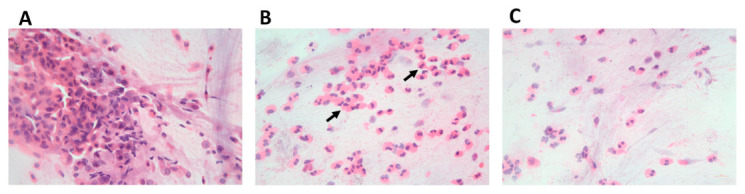
Eosinophils in nasal secretion smear changes during the study period. Representative photomicrographs of nasal mucosa with eosinophilic infiltration (black arrows). (Scale bar: 50 μm, original magnification, ×400). (**A**) Before treatment, (**B**) after 3 months of treatment, (**C**) after 6 months of treatment.

Mepolizumab significantly reduced SNOT-22 after 3 months of treatment and between T0 and T1 and T2. Also, VAS-NO, and VAS-rhinorrhoea showed a similar trend within the first 3 months of treatment. At 3 months of treatment, VAS-smell showed significant improvement. By 6 months, it had basically returned to normal or had mild symptoms (less than one point). VAS-facial pain showed a significant improvement only within the first 3 months (Figure 1). Furthermore, NPS significantly improved between T0 and T1, as well as eosinophils cells in nasal secretion sample (Figure 2). In the same study period, BECs showed a significant reduction between T0 and T1 and between T1 and T2 (Figure 2). A significant decrease in eosinophils was found in all the patients during 6 months of treatment (*p* < 0.001) (Figure 2). Eosinophils in nasal secretions changed during the study period (Figure 3). Refer to Table 2 for specific data.

Throughout the study period, all patients maintained their ongoing nasal treatment regimen, which included INCS and saline nasal irrigation. Mepolizumab was generally well tolerated throughout the 6-month study period. No serious adverse events were reported, and no patients required OCS or sinonasal surgery due to adverse events. All adverse events were mild and self-limited, and none led to treatment discontinuation. A summary of adverse events is presented in Table 3. The most frequently reported adverse event was injection site dermatitis (11/60 patients, 18.33%), followed by transient musculoskeletal pain (10/60, 16.67%) and fatigue (7/60, 11.67%).

## 4. Discussion

The real-life experience over 6 months confirms the efficacy of mepolizumab in patients with severe, uncontrolled CRSwNPs. Significant benefits were demonstrated as early as 3 months of treatment, with improvements across core efficacy measures including NPS, SNOT-22 score, and VAS scores for rhinorrhea and facial pain. By 6 months, further statistically significant improvements (*p* < 0.001) were observed in NPS, olfactory function, and eosinophil counts in nasal secretions. These findings suggest that mepolizumab may provide both early symptomatic benefit and progressive control of local type 2 inflammation, although longer follow-up is required to determine whether these improvements can translate into sustained disease remission.

Our findings are highly consistent with existing real-world evidence demonstrating that mepolizumab induces significant improvements in both patient-reported symptoms and objective inflammatory markers in the early phase of treatment [17], with sustained efficacy observed over time [6,17] and associated histological improvements in nasal polyp tissue, including reduction in eosinophilic inflammation and restoration of epithelial integrity [18]. The early onset of action coupled with durable clinical benefit holds substantial clinical relevance by enhancing patient confidence and promoting long-term treatment adherence. Notably, patients with higher baseline SNOT-22 score and those with comorbid asthma exhibit more pronounced biological responses to mepolizumab. This observation is further supported by a large real-world pooled analysis conducted by Schleich et al. [19], which included 1037 patients with severe eosinophilic asthma (with or without comorbid CRSwNPs). That study demonstrated that mepolizumab treatment was associated with a 30% incremental relative benefit in reducing clinically significant asthma exacerbations among patients with comorbid CRSwNPs compared to those without CRSwNPs. This finding underscores the importance of a holistic approach to managing type 2 inflammatory diseases, as treating one manifestation may yield synergistic benefits across comorbid conditions.

Recent evidence from real-world studies has provided insights into the comparative effectiveness of different biologics in CRSwNP [17,20,21,22]. A direct real-world comparison by De Santis et al. evaluated dupilumab, omalizumab, and mepolizumab in patients with severe uncontrolled CRSwNP, demonstrating that while all three biologics significantly improved quality of life SNOT-22 by 9 months, dupilumab exhibited the fastest onset of action in reducing NPS, with significant improvement observed as early as 4 weeks, whereas mepolizumab and omalizumab achieved significant NPS reduction only at 6 months [20]. Similarly, Ottaviano et al. noted that meta-analyses have indicated the superiority of dupilumab in symptom control and nasal polyp size reduction [17]. Additionally, Viskens et al. found that treatment with either mepolizumab or omalizumab demonstrated significant efficacy as early as 12 weeks, with the mepolizumab group showing a further reduction in NPS between weeks 12 and 24 [21]. These observations are consistent with a large US real-world study by Peters et al., which reported that dupilumab was the most frequently used biologic (89.8%) for CRSwNP, compared to mepolizumab (5.3%), omalizumab (4.8%), reflecting clinical preference [22]. However, these comparisons are primarily based on observational data, and ongoing head-to-head trials are expected to provide more definitive evidence [17,20,21,22]. In the present study, mepolizumab demonstrated consistent clinical improvement in the majority of patients, supporting its efficacy in this Asian population, although direct comparisons with dupilumab were not within the scope of this real-world exploration.

A distinctive contribution of our study is the direct documentation of significantly reduced local eosinophilic infiltration and ECP concentrations, assessed via cytology of nasal secretions, an observation less commonly captured in prior real-world cohorts. It has been established that mepolizumab is effective in type II driven CRSwNP regardless of BEC [19]. Inflammatory eosinophils preferentially accumulate in nasal polyp tissue rather than merely reflecting blood levels, and their local concentration correlates strongly with improvements in the sinonasal microenvironment [18]. These findings suggest that local eosinophils may serve as a more biologically relevant and clinically predictive biomarker than peripheral BEC.Rapid recovery of olfactory function represents another pivotal finding. VAS-smell scores improved significantly by month 3, with most patients approaching normal olfaction by month 6. This benefit likely stems not only from polyp shrinkage and consequent improvement in nasal airflow but also from structural restoration of the olfactory epithelium following inflammation resolution. Reduced local eosinophilia was accompanied by diminished mucosal edema, decreased goblet cell hyperplasia, and increased ciliated epithelial cell density [18,23]. Regarding safety, no serious adverse events were observed. Only mild, transient reactions, including fatigue, injection-site dermatitis, and musculoskeletal pain, were reported, consistent with safety profiles from other real-world studies. Notably, in patients with elevated baseline BEC, mepolizumab was associated with a lower incidence of adverse events compared with dupilumab [24]. None of the patients required OCS or repeat sinus surgery during the 6-month follow-up period, underscoring mepolizumab’s potential to reduce disease recurrence and alleviate the overall healthcare burden.

Several limitations should be considered. First, the modest sample size (*n* = 60), single-centre design, and six-month follow-up limit generalizability and preclude assessment of long-term outcomes (durability, relapse, safety). The limited enrollment was due to the recent approval of biologics in China, high out-of-pocket costs, and single-centre constraints. Second, the pre-post self-controlled design without a control group cannot exclude natural disease fluctuation or placebo effects; however, the consistent improvement in the majority of patients achieved a clinically meaningful response and large effect sizes suggest a genuine treatment effect. Third, the absence of sinonasal tissue biopsy limits mechanistic insights to surrogate markers (nasal secretions and blood). Fourth, concomitant treatments (INCS, nasal irrigation) were not standardized, and their individual contributions could not be analyzed. This was a single-arm observational study, so causal inference should be made with caution, and the findings should be considered hypothesis-generating rather than confirmatory. Despite these limitations, the study employed rigorous prospective methodology, standardized protocols, and objective outcomes, providing valuable preliminary real-world data in an Asian population after mepolizumab approval in China.

Future studies should build on these findings by enrolling larger, multi-centre cohorts with extended follow-up (beyond 12 months) to better evaluate long-term efficacy, recurrence risk, and cost-effectiveness. Incorporation of sinonasal mucosal biopsy is warranted to provide direct pathological evidence and enable a more comprehensive assessment of local tissue changes. In parallel, there is an urgent need to develop robust biomarker-based predictive models to accurately identify patient subgroups most likely to benefit, thereby facilitating truly personalized therapy. Furthermore, head-to-head comparative trials of different biologics in CRSwNPs are needed to clarify their relative efficacy and optimize treatment algorithms.

## 5. Conclusions

In summary, this real-world study offers initial evidence that mepolizumab may improve symptoms, polyp size, and olfactory function in severe uncontrolled CRSwNPs, possibly through suppression of type 2 inflammation. Due to the uncontrolled, single-arm design and modest sample size, these findings are hypothesis-generating and require confirmation in larger controlled trials.

## Figures and Tables

**Table 1:** Baseline characteristics.

Variable	Total (*n* = 60)
Age (years), Mean ± SD	40.5 ± 11
**Gender**	
Male	32
Female	28
**Smoking status**	
No	25
Yes	35
**Years with disease**	
<5 years	0
5–10 years	21
>10 years	39
**Comorbidities**	
Asthma	15
Aspirin intolerance	10
Airborne allergy	35
**Number of prior sinus surgeries**	
0	9
1	18
2	15
>2	18
**Structural nasal pathology**	
Septal deviation, valve pathology, septal perforation	15
**Biomarkers, mean ± SD**	
BEC (×10^9^/L)	459.51 ± 14.23
Nasal secretion eosinophils counts (/HP)	47.97 ± 3.02

**Table 2:** Analysis of VAS score and other data.

Variable	Baseline (Mean ± SD)	3 Months (Mean ± SD)	6 Months (Mean ± SD)	T1 vs. T0 (95% CI of Diff; *p* Value)	T2 vs. T0 (95% CI of Diff; *p* Value)
VAS-smell scores	8.22 ± 0.64	5.23 ± 0.74	1.15 ± 0.99	2.98 (−3.13 to −2.83); *p* < 0.001	−7.07 (−7.35 to −6.78); *p* < 0.001
VAS-NO	8.53 ± 0.95	4.70 ± 0.59	2.45 ± 0.62	−3.83 (−4.01 to −3.66); *p* < 0.001	−6.08 (−6.30 to −5.86); *p* < 0.001
VAS-rhinorrhoea	8.32 ± 0.95	4.92 ± 0.67	2.75 ± 0.63	−3.40 (−3.64 to −3.16); *p* < 0.001	−5.57 (−5.77 to −5.36); *p* < 0.001
VAS-facial pain	6.43 ± 1.03	3.42 ± 0.72	2.18 ± 0.72	−3.02 (−3.29 to −2.74); *p* < 0.001	−4.25 (−4.51 to −3.99); *p* < 0.001
SNOT-22 scores	69.10 ± 4.63	46.42 ± 4.32	22.10 ± 3.83	−22.68 (−23.28 to −22.09); *p* < 0.001	−47.00 (−47.85 to −46.15); *p* < 0.001
Nasal secretion eosinophils counts (/HP)	47.97 ± 3.02	32.13 ±2.13	18.35 ± 2.24	−15.83 (−16.09 to −15.57); *p* < 0.001	−29.62 (−30.04 to −29.19); *p* < 0.001
BEC (×10^9^/L)	459.51 ± 14.23	217.74 ± 19.27	169.73 ± 8.52	−241.77 (−247.20 to −236.34); *p* < 0.001	−289.79 (−292.68 to −286.89); *p* < 0.001
NPS	7.25 ± 1.02	5.57 ± 0.91	2.93 ± 0.69	−1.68 (−1.81 to −1.56); *p* < 0.001	−4.32 (−4.56 to −4.08); *p* < 0.001
ECP in nasal secretion (ng/mL)	457.87 ± 4.23	289.83 ± 2.76	60.76 ± 11.00	−168.04 (−168.50 to −167.58); *p* < 0.001	−397.11 (−399.66 to −394.57); *p* < 0.001

Note: All *p*-values were calculated using repeated-measure ANOVA with Tukey’s HSD post hoc test, all adjusted *p*-values are <0.001.

**Table 3:** Summary of adverse events during the 6-Month follow-up.

Adverse Event	No. of Patients (*n* = 60)	Incidence (%)	Severity	Relation to Treatment	Outcome
Injection site dermatitis	11	18.33	Mild	Definitely related	Resolved without intervention
Transient musculoskeletal pain	10	16.67	Mild	Possibly related	Resolved spontaneously
Fatigue	7	11.67	Mild	Possibly related	Resolved spontaneously
Serious adverse events	0	0	–	–	–
Adverse events leading to treatment discontinuation	0	0	–	–	–

Note: All adverse events were self-limited and did not require specific treatment. No severe or serious adverse events were recorded.

## Data Availability

The original contributions presented in this study are included in the article. Further inquiries can be directed to the corresponding authors.

## Abbreviations

BEC	Blood eosinophil counts
CRSwNP	Chronic rhinosinusitis with nasal polyps
ECP	Eosinophil cationic protein
ELISA	Enzyme-linked immunosorbent assay
ESS	Endoscopic sinus surgery
IL-5	Interleukin-5
INCS	Intranasal corticosteroids
NPS	Nasal polyp score
OCS	Oral corticosteroids
RCTs	Randomized controlled trials
SNOT-22	Sinonasal outcome test-22
VAS	Visual analogue scale
VAS-NO	Visual analogue scale score for nasal obstruction
VAS-facial pain	Visual analogue scale score for facial pain
VAS-rhinorrhoea	Visual analogue scale score for rhinorrhoea
VAS-smell	Visual analogue scale score for smell
